# Dementia and immigrant groups: a qualitative study of challenges related to identifying, assessing, and diagnosing dementia

**DOI:** 10.1186/s12913-018-3720-7

**Published:** 2018-11-29

**Authors:** Mette Sagbakken, Ragnhild Storstein Spilker, T. Rune Nielsen

**Affiliations:** 1 Department of Nursing and Health Promotion, Faculty of Health Sciences, OsloMet – Oslo Metropolitan University, Pilestredet 32, 0130 Oslo, Norway; 2 Norwegian Center for Migration and Minority Health, Norwegian Institute of Public Health, P.O. Box 222 Skøyen, 0213 Oslo, Norway; 3 Danish Dementia Research Centre, Rigshospitalet, University of Copenhagen, Section 6922 Blegdamsvej 9, DK-2100 Copenhagen, Denmark

**Keywords:** Dementia, Diagnosis, Diagnostic tools, Immigrant, Minority ethnic group, Health professionals

## Abstract

**Background:**

Along with the ageing of the general population, Europe’s migrant populations are also ageing, thus posing new challenges for dementia care services, particularly if the services are to be adjusted to persons with different linguistic and cultural backgrounds. From the perspective of health professionals, this study aims to explore challenges involved in identifying, assessing and diagnosing people with cognitive impairment/dementia who have different linguistic and cultural backgrounds.

**Methods:**

Research on health professionals experiences regarding the management of dementia among immigrants is scarce and qualitative methods was used to address the objective of the study. Using qualitative in-depth interviews and focus-group discussions, we sought to gather participants’ experiences regarding the diagnostic process for immigrants with dementia. The material was analysed and interpreted based on Kvale and Brinkmann’s descriptions of three different contexts of interpretation: self-understanding, critical common-sense understanding, and theoretical understanding.

**Results:**

Health professionals described how families could attribute symptoms of dementia to processes of normal ageing, while others saw the symptoms as something shameful; both instances delayed or hindered help-seeking. Many clinicians had limited experience with older immigrants suffering from dementia, and general practitioners (GPs) in particular experienced difficulties assessing dementia due to language barriers and difficulties related to the involvement of the family or an interpreter. The findings illustrate challenges in assessment, such as unfamiliarity with test situations among those being assessed and lack of knowledge regarding appropriate diagnostic tools among health professionals. Lack of continuity and poor information exchange in the chain of care seem to reinforce many of these challenges.

**Conclusions:**

Detection, treatment and care may be improved if primary care professionals strengthen their cross-cultural competences. Training in communication skills and in the use of cross-cultural assessment tools may help build competence and confidence when assessing and caring for people with different cultural and linguistic backgrounds. Closer collaboration among families, nurses in home-based services, dementia teams, and GPs may facilitate close monitoring of a patient over time. Such collaboration requires sufficient information exchange during transitions in the chain of care, continuity among health professionals, and a shared understanding of the goals for treatment and care.

**Electronic supplementary material:**

The online version of this article (10.1186/s12913-018-3720-7) contains supplementary material, which is available to authorized users.

## Background

Along with the ageing of the general population, Europe’s diverse migrant populations are also ageing, thus posing new challenges for dementia care services, particularly if the services are to be adjusted and made accessible to people with different linguistic and cultural backgrounds. Dementia has been declared as one of the major public health challenges of the century [1], and the global prevalence of dementia almost doubles every 20 years, with estimations reaching 65.7 million in 2030 and 115.4 million in 2050 [2]. The term dementia describes a wide range of symptoms associated with a decline in memory or other cognitive functions that is severe enough to impair a person’s ability to perform everyday activities. Other common symptoms include emotional problems and decreased motivation. Most dementia disorders, including Alzheimer’s disease (AD), are progressive in nature. Consequently, the need for support and care will increase over the course of the disease, often resulting in physical, emotional, and economic pressures, causing stress to families and caregivers and leading to increased societal costs [3, 4]. A recent literature review elaborating the benefits and challenges of early diagnosis [5] suggested that prompt evaluation of dementia allows potential detection and treatment of other causes of memory problems (e.g., depression and anxiety) and may reduce feelings of insecurity and anxiety in people with memory complaints and their families. Another argument is that a timely diagnosis will allow a person with cognitive impairment to make decisions regarding future living and care options/treatment, as well as financial and legal arrangements. Similarly, family and caregivers would be able to plan future family and/or public care and support [4, 5]. Lack of a diagnosis may also have consequences for a person with dementia, such that they do not obtain access to specialized dementia care (dementia teams, day care, and nursing homes specializing in dementia) [6]. Cognitive impairment can also result in poorer self-management of other chronic conditions, such as diabetes, heart failure, and hypertension [6]. Despite the advantages of a timely diagnosis and a general positive attitude towards screening programmes for dementia among older people in both the US and the UK [7], currently dementia screening seems premature because the aetiologies underlying dementia diagnoses are often uncertain, and treatment options for the main dementia disorders (e.g. AD) are limited [8].

To diagnose dementia, communication with patients and their caregivers is essential. In addition, physical and psychiatric examinations, observation of behaviours, assessment of activities of daily living, cognitive tests, blood samples, and brain scans should be performed for an accurate patient assessment [9]. However, knowledge among health professionals regarding dementia and proper assessment measures varies across and within countries, leading to a general underdiagnosis of dementia [4], especially at the early stages [10]. Even in high-income countries, only 50% of people living with dementia receive a formal diagnosis. In low- and middle-income countries, less than 10% of people living dementia are diagnosed [1].

Norway, compared with many other European countries, has a relatively recent history of immigration. In the last few decades, the country has experienced a significant increase from approximately 130,000 immigrant residents in 1987 to 916,000 immigrant residents in 2018. These people are either immigrants (746000) or are born in Norway to two immigrant parents (170000), constituting 17.3% of the total population [11]. Currently, the ten largest immigrant groups are from Poland, Lithuania, Somalia, Sweden, Pakistan, Iraq, Syria, Germany, Eritrea, and the Philippines. One-third of all immigrants have lived in Norway for less than five years, while 20% have lived in the country for at least 20 years. The groups that have been living in Norway for 20 years or more are mainly from Pakistan, Vietnam, Iran, Iraq, Eritrea and Ethiopia [11]. Between 2006 and 2015, work was the most common reason for immigrating to Norway. In 2015 and 2016, the influx of refugees increased, accounting for 29% of the immigrants in Norway [11]. Immigrants and Norwegians born to immigrant parents are, on average, much younger than the overall population, and only 9% are over the age of 60 years [11]. However, the number of individuals older than 67 years of age with an immigrant background is expected to increase tenfold to approximately 300,000 persons in 2050 [12]. This increase will not be due to a continued influx of new people but rather to the ageing of the young work immigrants who came to Norway in the 1960–1970s [12]. A literature review of health and access to health care among immigrant groups in Norway indicated that Norway needs to improve services by increasing the knowledge among health care personnel concerning the management of patients with different cultural and linguistic backgrounds. The review also showed that the patterns in health care use are different between immigrant groups and the native majority population and that immigrant groups face different challenges when accessing health care. This finding is particularly related to a lack of proficiency in the Norwegian language, which prevents immigrant groups from receiving important information from health-care personnel. Immigrant groups also lack knowledge regarding available facilities and consequently have poor access to health-care services [13]. Issues surrounding ageing and care for older people with immigrant backgrounds have received little attention in Norway. International research has shown ethnic differences in the use of dementia care services [14–16] as well as delays in access to diagnostic services for minority ethnic groups [17–19]. Studies have demonstrated low rates of anti-dementia medication prescription and use [20–25] and a reduced likelihood of entering a long-term-care facility [20, 25–27]. Additionally, relatively few older members from immigrant groups in Nordic countries live in care facilities, such as nursing homes [28]. One register-based study from Norway found that the proportion of persons from immigrant groups in Norway, especially those from low- and middle-income countries, who received a diagnosis of dementia or memory impairment in primary health care, was significantly lower than that of ethnic Norwegians [21].

As in other European countries, Norwegian dementia care policies focus on the importance of timely diagnosis and follow-up when cognitive impairment and dementia is suspected. In the second action plan on dementia from the Ministry of Health and Care Services, “Dementia Plan 2020”, the challenges encountered by immigrants in accessing and using services are emphasized, as well as the need for training and guidance for health-care personnel [29].

Therefore, the aim of the present study was to explore the challenges involved in identifying, assessing, and diagnosing persons with different linguistic and cultural backgrounds from the perspective of health professionals. The present paper is part of a larger study on older immigrants and dementia in Norway with the overall goal of assisting the Norwegian Directorate of Health in designing appropriate strategies for the care of these groups. The target groups for this project are immigrants aged 50 years or older, family caregivers, health professionals, and decision- and policy-makers. The main findings are presented in a Norwegian report [30], and an article addressing family care patterns has been published elsewhere [31]. In addition, other articles are in the process of being published.

## Study setting and methods

### The research context

Norway has a tax-financed public health-care system, and equal access to health care is in principle available to all residents. The maximum fee for health services is 230 euros per year (excluding dental care). All persons registered in Norway (including all immigrants who are legal residents) are allocated a personal identification number and have the right to choose their own general practitioner (GP). The Norwegian health care system is semi-decentralized, implying that the state is responsible for specialist care (administered by four Regional Health Authorities), and the municipalities are responsible for primary care. Public sources account for more than 85% of the total health expenditure, and most private health financing comes from household out-of-pocket payments. The municipalities are responsible for primary health-care and social services, providing assistance for older persons receiving day care or care at home, or for those living in nursing homes [32]. GPs act as gatekeepers to secondary care (hospital care being free of charge) and are expected to use their best professional judgement to secure effective and fair allocation of resources, implying that they must distribute resources between patients with competing needs [33]. Immigrants lacking proficiency in the Norwegian language are entitled to an interpreter during medical encounters (free of charge), a right that is emphasized in the National Strategy on Immigrants’ Health [34]. Norway has one of the highest physician densities in Europe but still struggles to ensure geographical and social equity in access to health care. Norway’s 5.2 million inhabitants are dispersed over nearly 400,000 km^2^, which may explain some of the challenges [32]. Nearly 80% of the 422 municipalities in Norway have established dementia teams and/or dementia coordinators for evaluation and follow-up, and one in three has both [29]. A patient’s GP or medical doctor at a nursing home is responsible for assessment and diagnosis. However, cognitive testing and assessment can be conducted in the home by a dementia coordinator in close collaboration with nurses in home based services and the GP. In the Norwegian Dementia Plan 2020, collaboration between the medical practitioner and dementia teams/coordinators and other health and care personnel is strongly recommended [29]. However, many nursing-home residents and people living at home show signs of cognitive impairment but have not been assessed for dementia. A recent study found that up to 50% of nursing home residents with definite signs of dementia had not been diagnosed [35]. One of the priorities of the Dementia Plan 2015, Norway’s first action plan on dementia, was to develop and test models for the assessment and diagnosis of people with dementia. A programme called ‘Guide for Dementia Assessment in Primary Health Care’ was developed, evaluated, and revised during the plan period, and its use is recommended [36].

### Design

Research examining the views and experiences of dementia among older immigrants in relation to health professionals, patients, and relatives is scarce, and the research team considered qualitative methods the most appropriate approach to address the objective described in the introduction. Through qualitative individual and dyad interviews as well as focus group discussions, we sought to gather participants’ own experiences and perceptions regarding the treatment and care of people with cognitive impairment/dementia with an immigrant background.

### Sample strategy

We conducted a purposeful sampling of 27 health professionals, including 18 women and 9 men, who represented different geographical parts of Oslo as well as Norway, including six different counties (Troms, Buskerud, Rogaland, Sør-Trøndelag, Oslo, and Akershus) in the Northern, Western, and South-Eastern parts of the country. To identify participants who had actual experience with older immigrants with dementia/cognitive impairment, we conducted a systematic and extensive recruitment process in immigrant-dense areas. We applied a combined approach in which we sent an information letter about the study to all the GP centres in four districts of Oslo with large immigrant populations and to existing networks and contacts within the field. During recruitment in other parts of Norway, we called or sent informational letters (followed by phone calls) to relevant persons and institutions. Based on their responses, most of the people contacted clearly did not have experience in assessing and diagnosing older people with cognitive impairment and an immigrant background.

Given that different health professionals are involved in different stages of the diagnostic process, the final sample comprised health professionals from diverse professional backgrounds (see Table 1). Thus, our sample consisted of medical doctors, nurses, dementia coordinators, and other relevant/experienced health care professionals working in primary and secondary health care. The participants worked in GP centres, nursing homes, short-term nursing homes, day-care centres, home-based services, geriatric polyclinics, psychiatric polyclinics, hospital-based memory clinics, a community health centre with services for refugees, or were part of a dementia team (teams of health professionals working in primary care and assisting GPs in dementia diagnostics). The participants represented health professionals originating from seven different countries in addition to Norway.

**Table 1:** ᅟ

Interview form	Participants	Workplace	Professional background	Country background	Number
Individual interview	1 woman	City district Dementia coordinator	Registered nurse (1)	Norway	1
Individual interview	1 man	Memory clinic	Medical doctor, specialist in geriatrics (1)	Sweden	1
Individual interview	1 woman	Short-term nursing home	Medical doctor (1)	Norway	1
Individual interview	1 man	Nursing home/Long-term care facility	Medical doctor (1)	Norway	1
Individual interview	1 man	General practitioners’ office	Medical doctor, specialist in general practice (1)	Norway	1
Individual interview	1 woman	General practitioners’ office	Medical doctor, specialist in general practice (1)	Pakistan	1
Dyad interview	2 men	Nursing home/Long-term care facility	Registered nurse/head manager (1) Medical doctor (1)	Norway	2
Dyad interview	2 women	Refugee health service	Community health / public health nurses (2)	Norway	2
Dyad interview	1 woman, 1 man	General practitioners’ office	Medical doctors, specialists in general practice (2)	Norway, Pakistan	2
Focus group discussion	4 women	Geriatric and psychiatric polyclinic	Registered nurses (2) Registered nurses, specialised in geriatrics (2)	Norway	4
Focus group discussion	4 women, 1 man	Homebased services/dementia team	Auxiliary nurses (2) Registered nurses (2) Occupational therapist (1)	Norway	5
Focus group discussion	4 women, 2 men	Nursing home	Auxiliary nurses (3) Social worker (1) Registered nurses (2)	Ethiopia, Sri Lanka, Morocco, Philippines, Pakistan and India	6
*Total*	*18 women, 10 9 men*		*Registered nurses (7)* *Registered nurses, specialised in geriatrics (2)* *Community health / public health nurses (2)* *Medical doctors, specialised in general practice (4)* *Auxiliary nurses (5)* *Medical doctors, working in nursing home (3)* *Medical doctors, specialised in geriatrics (1)* *Registered nurse/head manager (1)* *Occupational therapist (1)* *Social worker (1)*		*27*

### Use of terminology

In this manuscript, we use “minority ethnic groups” when we refer to studies from outside Scandinavia. However, in the parts of the manuscript where we refer to Norwegian statistics, Norwegian/Scandinavian studies, and the present project, we use the term “Immigrant”, which is the terminology used by Statistics Norway and Statistics Denmark (additionally, “foreign-born” which is the most prevalent concept used in Sweden). In Norway, immigrants are defined as “Persons born abroad of two foreign-born parents and four foreign-born grandparents” (11) and is the most commonly used term in academic and public discourse. Importantly, the use of “Immigrant” also indicates that we are not referring to our native population, the Saami’s, or minority ethnic groups such as the Kvens, a group that started to migrate to Norway (Finish origin) as early as the 1500s.

### Data collection

We conducted seven in-depth interviews (IDI), three dyad interviews (two health professionals at the same time), and three focus-group discussions (FGDs) with different health professionals at the workplaces of the participants. Both the IDIs and the FGDs lasted 60–90 min. In-depth interviews, including 1 dyad interview with 2 GPs working together in a district outside Oslo, were performed with all the GPs, doctors in specialized care, and one of the experienced dementia coordinators – i.e., those who could provide an in-depth account of the diagnostic procedures/assessment circumstances. The FGDs consisted of 4–6 participants and were conducted with nurses (including representatives of dementia teams), auxiliary nurses, and other relevant personnel with roles in the diagnostic processes. By conducting FGDs, we were able to explore different views and experiences, e.g., in regard to collaboration with doctors (in primary and specialized care). Through method and respondent triangulation, we elucidated complementary aspects of the same phenomenon by approaching topics in depth through in-depth individual interviews and by inspiring new associations and perspectives through the focus groups discussions.

Two semi-structured guides (see Additional file 1, Additional file 2) based on a literature review, preceding FGDs with healthy older immigrants, and exchanges of experience with experts in the field, was developed to address the objective of this particular study. However, the guide was flexible, in that the interviews and FGDs were governed by answers and themes introduced by the participants, and the guides were adjusted to a certain extent to fit the profession or position held by the participant(s). The aim was to gather participants’ perspectives and experiences related to their specific workplaces and responsibilities and to interpret the meaning of these findings.

### Recruitment and informed consent

Participants were informed about the study through phone calls or e-mails and written study information. In the case of nurses and nurse assistants, the head of the department was involved in the recruitment process, while the doctors were contacted directly. Participation in the study was coordinated by telephone or email. Written informed consent was obtained from all participants.

### The research team

The research team consisted of three researchers with different backgrounds. The first author (MS) is a registered nurse and has a PhD degree within the field of global health, inspired by medical anthropology. At the time of the study, she was the research leader of The Norwegian Centre for Migration and Minority Health (NAKMI). The second author (RS) is a registered nurse with an MPhil in Health economics, policy and management and is employed as a Senior Adviser at NAKMI, Norwegian Institute of Public Health. The last author (TRN) has a background in clinical neuropsychology and currently holds a research position in the Danish Dementia Research Centre, Rigshospitalet, University of Copenhagen, and is in his work drawing on a bio-psycho-social understanding of dementia.

### Analysis and interpretation of the text

The material was analysed and interpreted based on Kvale and Brinkmann’s [37] descriptions of three different contexts of interpretation: 1) *self-understanding*, 2) *critical common-sense understanding*, and 3) *theoretical understanding*. Kvale and Brinkman define self-understanding as a condensed form of what the subjects themselves understand to be the meanings of their statements. Critical common sense understanding may include a wider frame of understanding than that of the participants themselves. At the theoretical understanding level, a frame is used for interpreting the meaning of a statement. These interpretations go beyond those that are based on common sense.

To optimize the analytical process and to achieve a systematic approach to the data material, we worked with each interview immediately after the interviewing session. The tape-recorded interviews were transcribed to access the material as presented by the participant (self-understanding). Then, the researcher wrote down a first impression and reflections upon the interview, followed by a more detailed reflection log consisting of descriptive and analytical notes (critical common-sense and theoretical understanding). Based on experiences, reflections, and identification of novel topics that emerged from single interviews, the interviewing guide was constantly adjusted. The first step of the coding process involved two of the co-writers who read the transcribed interviews several times to gain a sense of the whole. After separate, in-depth readings of the transcripts, meaning units that derived from the data were identified by colour coding in order to structure the participants’ utterances. This process involved searching the entire material for similar and contrasting statements. After several discussions related to which themes were represented by each unit of meaning, the researchers formulated the subjects’ self-understanding in a condensed form. In the next step of this iterative process, further attentive reading and discussion uncovered nuanced meanings related to the initial meaning units, and the interpretation was enriched by the addition of general knowledge about the content of the subjects’ statements. In the last step, the different sub-themes were linked together and described in four central themes that reflected the focus of the study. For example, sub-themes such as unfamiliarity with test situations among those assessed, use of inadequate diagnostic tools, language difficulties, and use of an interpreter were linked together and formed the theme *challenging assessment situations*. This more comprehensive interpretation included contextualizing the critical common-sense understanding by using theoretical frameworks and previous research, thus advancing our analysis to a higher level of abstraction. Therefore, the third context of interpretation is reflected in the discussion.

### Validity

The use of triangulation served to strengthen the validity of the data. By triangulating sources (different health-professionals), health-care settings (e.g., GP centres, nursing homes, day-care centres, home-based services, geriatric and psychiatric polyclinics, and hospital-based memory clinics), the time points in the diagnostic process (experiences related to different points in time in the overall assessment process), methods (FGDs and IDIs), and analysts (two researchers reading and analysing all the transcripts), we examined variations and contradictions as well as the consistency of different data sources. Even though all the researchers are health professionals, the different positions as well as educational background facilitated a nuanced discussion of how to interpret the data. Theme saturation was reached due to the triangulation in the study, as reoccurring themes were discussed and validated with different participants representing different experiences in different parts of the assessment situation.

## Results

Four main themes were identified during the analytical process. These themes were *delayed help-seeking*, *health professionals lacking experience*, *lack of knowledge and use of appropriate diagnostic tools*, and *challenging assessment situations*. These themes are elaborated below.

### Delayed help-seeking

Many of the participants mentioned that based on their experience, strong norms related to family care could lead to a lack of or a delay in diagnosis among older patients with an immigrant background. Health professionals also described how some people could attribute symptoms of dementia to the processes of normal ageing and consequently fail to seek or delay seeking medical help, while others associated the symptoms with shame, often associated with bad karma or a punishment from God. A GP relayed the following:

“Either it (the response) can be a form of belittling in a way, or they relate it to Gods will … and then we are not meant to do anything about it.”

Another topic that was addressed was how family members around the patient would compensate for the symptoms with a more collectivistic approach to support and care. A GP elaborated the following:

“In many of these cultures, a lot of people are always around, and memory is somehow a shared thing, meaning that you (family members) will compensate more when it fails.”

In the above quote, the role of the extended family is accentuated. The GP indicates that memory loss may be considered a normal part of ageing, and compensation for an older family member’s memory problems may be considered a normal part of family life, potentially making symptom recognition more difficult. Both nurses and GPs relayed difficulties in assessing patients living at home or accompanied by family members since family members often answer on behalf of the patient or help and correct the person being assessed.

Even though strong family involvement was portrayed as the most common pattern, nurses and GPs also emphasized that some older immigrants live alone (e.g. due to separation from family members during the migration process) and often have different types of social problems, without relatives available to notify health professionals about concerns regarding the cognitive status of a person. A nurse in home-based services elaborated the following:

“…many come late… because ... they do not come by themselves, it may be neighbours that have provided notes of concern… because so many people live alone. […] many have social problems, and many do not have relatives […] and a lot of shame exists around this… […] So you may end up offering help at a very late stage, and the situation can be very difficult…”

Therefore, many different factors may influence and delay help-seeking and subsequent access to care and support. Due to different compensation mechanisms or perceptions, such as viewing symptoms of cognitive impairment as a “normal part of ageing” or as something shameful (not to be exposed), the affected person may suffer from progressive or advanced symptoms by the time contact with health services is initiated. Another nurse elaborated the following:

“…some may provide a note of concern, and then the situation is already serious and complicated, and when they do not want to receive help at that point, then you need to bring in the GP, and then it is […] this responsibility they have in assessing the ability to consent and … some just will not touch it…”

As illustrated in this example and in several other interviews with nurses in primary health care, a GP may be reluctant to assess patients with a different language or culture. This phenomenon seems related to general uneasiness in approaching “complicated cases”, including challenges related to assessing the ability to consent in persons with advanced symptoms and a reduced or no ability to speak Norwegian.

There were also some examples of close and positive collaboration between home-based services, GPs, and secondary services. In such cases, the dementia teams seemed to play a key role. A dementia coordinator spoke of her experiences as follows:

“Often, we contact the GP and try to establish a collaboration […]. We have been participating in home visits among patients who do not speak Norwegian very well… and we also attended a course in RUDAS…” (a cognitive screening instrument designed to minimize the effects of cultural learning and language diversity when assessing cognitive functioning).

Many advantages was described regarding the use of dementia teams; these advantages include performance of (part of) the evaluation in a patient’s home over several visits and in close collaboration with a GP and home-based services. Several participants mentioned the need for further utilization and integration of dementia teams/coordinators in primary health care. In primary health care, the importance of continuity was emphasized, particularly among the nurses. Lack of continuity in interactions with older people with different cultural and linguistic backgrounds was described as a barrier to getting to know a person and monitoring his/her cognitive development over time.

### Health professionals lacking experience

One of the main findings was that although the participants worked in areas with a high density of immigrants, many had limited experience with older immigrants with cognitive impairment or dementia and experienced difficulties related to the need to involve family and/or an interpreter. The participants described consultations in which they lacked confidence in terms of how to communicate suspected cognitive impairment, and some GPs mentioned that they missed concrete signs, such as family members expressing concern regarding their relatives’ driving skills. Discussing driving-licence status was a common and concrete strategy for addressing symptoms of cognitive impairment among older persons with an ethnic Norwegian background, while older immigrants often do not possess a driver’s licence. Limited experience combined with language barriers seemed to create a feeling of insecurity and hesitation in managing this group of patients, and most of the participants, including the hospital-based specialists, emphasized the need for increased competence in responding to the needs of immigrant patients with dementia. A nurse from home-based services spoke of her experience as follows:

“A lot of GPs refer directly to geriatric departments and policlinics even if cognitive impairment is obvious because they…. they may feel that they do not have the competence […]. We sometimes suggest collaboration (between nurses in home-based services, GPs, and dementia teams), but in cases where a language barrier exists, they say that the patient must be dealt with by specialist services.”

Several sources, including some GPs, confirmed that GPs often refer directly to a specialist without trying to perform a clinical assessment themselves. One GP who preferred to refer directly to specialist services relayed the following:

“It's difficult to reveal I think. Well ... yes. I do not have that many older immigrants on my list, at least not who have developed cognitive impairment so clearly that you can recognize it in a way. I have someone, but not very many (patients). […] They have received an assessment in the hospital…”

Another GP explained:

“Handling an interpreter as well as all the other factors involved is so difficult, so examining a person in a specific, geriatric ward in the hospital is advantageous.”

By referring to the complexity of cases and their limited experience with dementia, the GPs rationalized direct referral to a specialist and thus circumvention of all the insecurities involved in the consultation.

### Lack of knowledge and use of the appropriate diagnostic tools

Another important finding was related to the lack of knowledge and experience with appropriate diagnostic tools. Most of the health professionals, excluding some specialists and two GPs, had not heard of, and only a few had ever used, The Rowland Universal Dementia Assessment Scale (RUDAS) [38], a short cognitive screening instrument designed to minimize the effects of cultural learning and language diversity when assessing general cognitive functioning. Most GPs and some specialists used the common Mini-Mental State Examination (MMSE) [39] and the Clock Drawing Test [40] as standard screening tools. According to an experienced representative of one of the dementia teams, GPs that use these standard tests are often convinced of the validity of the tools, as demonstrated by the following:

“If they (GPs) have not really made an effort to understand how to use these tools, then they tend to think that these tests are fool-proof….”

Some of the specialists and one of the experienced GPs emphasized that many of the questions in standard cognitive tests are culture-specific and require knowledge related to Norwegian culture and history, understanding of nuances in the Norwegian language, as well as a general level of education. A specialist in the field elaborated the following:

“I have often wondered about people with a foreign background that have been assessed with tests that we know are language-specific; for example, so-called verbal intelligence tests focus on the understanding of words, expressions, proverbs and so on, all of which are specific for the language that they are constructed within. […] One of the interpreters that I work closely with told me that he was to interpret for a man from Nigeria, and at the neuropsychologist (referring to dementia assessment), the man received the question: ‘What do you associate with the word “Kaupang?”’ (Historical word/place based on Norse mythology).”

The specialist cited above, along with a GP who is highly experienced in diagnosing dementia, described several middle-aged people with immigrant background who were misdiagnosed with dementia due to the use of screening tools that were not adjusted for relevant cultural, language, and educational factors and were therefore used improperly in their opinions.

### Challenging assessment situations

The findings revealed specific challenges involved in assessment situations and in consultations at clinics where specialists assess cognitive impairment daily. Some of the most experienced participants indicated that unfamiliarity with the test situation rendered conventional cognitive testing inappropriate because many of the people undergoing assessment lack previous experience with test situations (similar to those in school), and they are therefore unable to understand the situation and to motivate themselves to perform well. Another issue raised by a specialist along with some GPs was the difficulty in determining the expected level of cognitive performance from people who are illiterate, have a low level of education, or had a different type of schooling. A GP elaborated the following:

“…when you test people, you obtain a result that shows a score or something, right? And then you have to relate it to something…. And of course, you cannot compare someone who has attended 4 years in a Koranic School in a village in rural Pakistan and someone like me… […] you have to compare the results with someone similar… ”

In addition, all the GPs and the nurses working in dementia teams discussed the challenges associated with using interpreters during an assessment situation. Some emphasized the vulnerability associated with a situation with an unfamiliar interpreter present or a situation that could be unfamiliar, scary, and humiliating. They had also encountered a general concern among patients and their relatives that confidentiality would not be maintained. Others emphasized problems with interpreters changing sentences or meanings to facilitate the translation and thus introducing errors in the assessment situation. A specialist in diagnostics relayed the following:

“…these interpreters are in a test situation, which not only requires mediating the meaning of what the patient says, which is what you normally do…” But here, I also want to know how the patients are actually saying it, whether they have difficulties in expressing themselves, pronouncing words correctly, or whether they say strange things that are difficult to understand… […] or if I give a patient a task to solve and they do it wrong, and then the interpreter says “no, this is not right!”

The challenges with interpreters are also related to the fact that an assessor must often use unfamiliar interpreters and that interpreters may not be trained to translate in these kinds of situations.

Although specialist services often appear to assume much of the responsibility in diagnosing people with different linguistic and cultural backgrounds, one GP raised a concern regarding how the dementia assessment process ended for patients with an immigrant background if they were admitted to a nursing home as follows:

“Those who are in the process of being referred from geriatric out-patient departments or those undergoing assessment … sending them for further assessments and controls can be relevant […] We do confer … but more often they (specialist services) call us (the GP) and say ‘we hear that this person has received a permanent nursing home offer, so the assessment does not need to continue.’”

Ending the diagnostic process due to a person’s admission to a nursing home can likely delay or jeopardize the possibility of receiving an aetiological diagnosis and thus affect the quality of treatment and care.

## Discussion

The present data highlight a number of barriers to early and correct diagnosis of cognitive impairment/dementia in persons with diverse cultural and linguistic backgrounds in Norway. One of the barriers is related to health professionals’ interpretation of cultural norms or preferences for family care, thus preventing or delaying health-seeking behaviour, diagnosis, and treatment and care; these findings are similar to those of several other studies [41–43]. Studies focusing on care patterns among minority ethnic groups often emphasize strong identification and solidarity with family members, both nuclear and extended [44]. The most frequently examined aspects of cultural values and traditions are those of *familism* and *filial piety* [44]. The concept of familism refers to strong identification and solidarity with family members, and involves strong normative feelings of attachment, responsibility, and reciprocity. The concept is sometimes interpreted as contradictory to the Western value of individualism. Filial piety, a fundamental value in Confucian (Chinese) ethics, reflects respect for parents and older persons and placing families’ needs over individuals’ needs. Filial piety is a key value in Asian cultures, but similar ethical values and norms are parts of many other cultures and religions, including Western-oriented cultures [44]. A meta-analysis of caregiving patterns among different ethnic groups found that filial piety was more prominent among minority ethnic groups [45]. Other review studies focusing on attitudes and experiences of caregivers of people with dementia among black and minority ethnic groups from the USA and UK found that many family members view caring for a relative with dementia as a normal part of life, reflecting a natural and extended role in the family [41, 46]. This perspective is explained by the fact that seeking help from the outside is considered disrespectful of a person’s autonomy or reflects failure to fulfil one’s familial obligations. However, as underlined in the meta-analysis of caregiving patterns [45], there is no evidence that minority ethnic caregivers in general rely less on formal support than do Whites because of differences in value systems. For example, structural barriers like language problems or differences in acculturation, potentially related to education and socio-economic status, may explain why some groups of Asian caregivers use these services less than Whites [45]. Thus, there may be huge differences both between and within immigrant groups, and it is important that health professionals become aware of the danger of making generalized and stereotyped assumptions about older immigrants with dementia and their families [46]. In other studies, family care and delayed health-seeking behaviour are explained using descriptive models showing that symptoms of cognitive impairment are accepted as normal memory problems in old age [47] or showing the stigma and shame associated with these types of symptoms [48–50]. These views have also been reported in immigrant groups in Scandinavia [51]. A recent Lancet review indicated that later diagnosis is a particular problem for those from minority ethnic groups in which stigma, lack of understanding that dementia is an illness, and lack of acceptance of medical care are inherent [52]. However, within this discussion it seems important to acknowledge that the term dementia is a Western construct, and what is considered as normal/abnormal aging is a constructed one. Further, the understanding of the aetiology of dementia is constantly changing, and what may have been portrayed as “incorrect” lay conceptions of dementia in earlier research may correspond well with how dementia is understood in the Western part of the world today. For example, updated research shows that lifestyle factors such as depression, physical inactivity and social isolation are risk factors for the development of dementia in later life (52); factors corresponding well with many “lay conceptions” of understanding.

Another issue that causes a delay or a lack of diagnosis and care according to our study was that some of the older immigrants encountered by nurses and GPs lived alone, removing the common element of family care patterns. Notably, changing demographics may influence families and their ability to provide care; therefore, not all people from minority ethnic groups are cared for by their families [53]. For immigrants from low- and middle-income countries, the shift from a high-mortality and high-fertility society to a low-mortality and low-fertility society has caused an increase in the actual number of living generations and thus a decrease in the number of relatives that can live together with their extended families [53]. Additionally, immigrant families may be separated or divided during the migration process, which can make ‘traditional’ care difficult to provide in an extended family context. Lastly, the emergence of new roles as well as competing demands associated with the roles of different family members in new settings [53] may result in challenges in allocating time to care for family members who become severely ill.

The findings from the above-cited studies indicate that patients with different linguistic and/or cultural backgrounds, besides potentially holding values in accordance with those of *familism* and *filial piety,* may face barriers due to changing demographics as well as structural barriers in receiving diagnostic assessments and accessing care. Further, as shown in our findings, complicated cases with language- or culture-related barriers, sometimes with an additional barrier related to the ability to consent, may cause GPs to be reluctant to pursue such cases. Although the GP or the medical doctor at a nursing home is responsible for evaluation and diagnosis (in close collaboration with dementia coordinators and other relevant health professionals) [29], they may generally have limited experience with dementia diagnostics compared to specialist departments; and our findings show that GPs prefer to refer patients directly to a specialist without performing a clinical assessment on their own. In a systematic review and meta-analysis of ethnic differences regarding dementia treatment, care, and research, consistent evidence, mostly from the US, demonstrated that people with minority ethnic backgrounds access diagnostic services later in their illness. Once they received a diagnosis, they were less likely to receive anti-dementia medication, participate in research trials, and receive 24-h care [25]. In nationwide Danish studies examining the quality of diagnostic dementia evaluation, the use of anti-dementia medication, and nursing home-based care for patients with a minority ethnic background compared with the general population, significant disparities were also evident [20, 54]. One of the hypotheses of the authors is that patients from minority ethnic groups receive poorer treatment due to a more nihilistic approach towards these patients [9]. Although no Norwegian studies have examined the quality of the diagnostic process, a register-based study compared the proportions of ethnic Norwegians and immigrants with a diagnosis of dementia or memory impairment in primary health care and investigated patterns of utilization of primary health-care services and the use of pharmacological treatment. This study found that a significantly lower proportion of immigrants, especially those from low- and middle-income countries, had received a diagnosis of dementia or memory impairment. Additionally, among the patients with these diagnoses, anti-dementia medication was purchased 20–50% more often by ethnic Norwegians than by immigrants [21]. Some explanations for these significant disparities are likely to be found in our study. A common strategy or topic to initiate a discussion regarding cognitive impairment (i.e. driving licence) was often not available, and communicating with family members during assessment situations can be difficult. The participants described various barriers, such as difficulties in communication and therefore also in assessment of cognitive impairment in persons with diverse linguistic and cultural backgrounds. As frequently reported in the literature [55–57], the need for interpreters further complicated assessments because they often lack competence in interpretation in assessment situations and may change sentences or their meaning to facilitate the translation.

The findings of our study also provide examples of patients who were referred for or were undergoing a dementia assessment that was terminated if they were admitted to a nursing home. Determining whether this practice is specific to immigrants or whether similar patterns exist among ethnic Norwegians is difficult. However, considering the findings reported by Diaz et al. [21], the quality of the diagnostic process (including the likelihood of receiving anti-dementia medication) may be poorer for immigrant groups than for ethnic Norwegians. Several review studies regarding minority ethnic groups and dementia indicate that the service needs of these groups have not been identified and therefore remain unmet, representing another explanation for the under-utilization of services [41, 58, 59].

However, under-utilization of services may not be specific to dementia since people with an immigrant background generally face more barriers in accessing health services than does most of the population, especially the oldest people [60]. These barriers may be due to factors such as health illiteracy, lack of interpreter services, communication problems, different health beliefs, negative experiences with health care services, or high direct or indirect costs [61–63].

Another important finding of our study is related to the prevalent use of standard cognitive tests as many of the questions, e.g. those in the MMSE, require knowledge of Norwegian language and geography as well as a general level of education [64, 65]. In a survey conducted in clinical dementia centres in 15 European countries (all receiving minority ethnic patients on a regular basis), 64% described diagnostic evaluations of patients from minority ethnic groups as challenging for reasons such as communication problems and lack of adequate assessment tools [54]. However, as mentioned by some of the most experienced participants in our study, the challenge in achieving good test situations is not only related to language or a lack of valid tools. For example, tests in which maximum scores are desired may not be appropriate for patients who are unfamiliar with similar test situations. People without a formal education are typically not “test wise”; that is, they are not familiar with being tested and may not know how to behave during a testing situation [66]. Another problem is that assessors may not know what level of performance to expect from people who are illiterate or received a different type of schooling. Although the RUDAS, a brief screening test for cognitive impairment in multicultural populations that was developed as an alternative to the MMSE, is available in Norway, it is neither mentioned in the Norwegian Dementia Plan 2020 [29] nor recommended in the Guide for Dementia Assessment in Primary Health Care [36]. Since the assessment tool is not part of the official recommendations for the evaluation of dementia, this may explain why it is not frequently used in clinical practice. The RUDAS is supposed to be easy to use in primary health care, and use of the test implies that the interviewer encourages the test-person to speak his/her dominant language; it has been administered in more than 30 languages without a need to change the wording or adapt any of the items [38]. Unlike other tests, this test is also reliable when using an interpreter. According to the primary reference, the total score is affected by age, but not by education or language [38]. However, this feature has been challenged by more recent studies that observed an effect of education in people with very limited education [67]. Using tools that have only been validated in a Western context obviously creates sources of error [68], especially if the clinicians performing the assessments do not collaborate closely with home-based services or dementia teams that can observe a patient in other settings. A study investigating the complexity of cognitive assessment of older persons in British minority ethnic groups found false-positive results using the MMSE for 6% of non-impaired white people and 42% of non-impaired black people. The authors concluded that current cognitive tests under-estimate abilities in black and minority ethnic groups and that adjusting tests and other existing services may lead to improved outcomes for people with dementia and their caregivers [69].

A systematic review of trials to increase dementia diagnostic rates found no clearly successful single intervention [70]. However, a more proactive approach in primary care [71], in which patients or their families are asked about concerns regarding memory (when suspected) and how they potentially want to proceed, may pave the way for adjusted treatment and care. To facilitate such an approach, increased continuity and improved collaboration among professional care providers involved in the chain of care for persons with dementia from minority ethnic groups are needed. The participants described varied patterns of collaboration; some groups of health professionals collaborated well, whereas others reported little collaboration and interrupted communication in the chain of care. The participants in our study suggested that dementia coordinators should be utilized even more consistently to facilitate collaboration. In the early stage of the disease, registered nurses working in primary care and/or dementia coordinators can bridge the gap between specialist care in hospitals and care services in municipalities.

### Limitations of the study

By triangulating sources (different healthcare professionals and healthcare settings) and methods (FGDs and IDIs), we examined variations and contradictions as well as the consistency of different data sources. However, the topic is addressed by approaching only health professionals and other themes would likely have appeared if we had included patients and their relatives. In addition, we cannot rule out the possibility that the different methods used with different healthcare professionals may have affected the content of the interviews. The aim of the present study was to explore the challenges faced by health professionals in identifying, assessing, and diagnosing persons with different linguistic and cultural backgrounds. Since the number of older immigrants in Norway is still rather small and the experiences of health professionals with patients with dementia and an immigrant background is very limited, it would not have been fruitful or even possible to focus our study on specific immigrant groups. The terminology and classification of ethnicity and minority ethnic groups are not agreed on internationally. For the purpose of this study, we used the term “Immigrant”, which is the terminology used by Statistics Norway. However, by asking health professionals about their experiences with immigrants in general, there is a risk for generalisations and simplifications and that the heterogeneity of immigrant populations are obscured. The conclusions should be read with care as it may conceal the possible interplay of different variables. Further research is needed to better understand different needs of people from different immigrant communities.

## Conclusion

As suggested by the findings of our study and supported by the World Alzheimer Report 2016, timely diagnosis and intervention in dementia relies on collaboration between patients, family members, and health professionals, between primary care services and specialist services, and between interpreters and those who assess patients. Viewing dementia diagnostics as a process that involves several partners, continued communication, and coordinated follow-up requires acknowledgement of the many potential advantages, such as the resultant strategies for those affected, their families, and service providers. Although screening programmes for dementia are unwarranted at this time, primary care professionals, including GPs, may need to be convinced of the importance of a timely dementia diagnosis if changes in clinical practice are to occur. Detection and subsequent treatment and care may be improved if primary care professionals gain more cross-cultural competence, including experience with the use of cultural sensitive tools, and are generally more involved in the diagnosis, treatment, and care of people with dementia. Although not specifically investigated in the present study, the identified challenges will probably affect all steps along the dementia care pathway, including but not limited to giving advice about risk reduction, participation in interventions after diagnosis including post-diagnostic support, palliative care and interventions for challenging behavior. Training in communication skills and in the use of cross-cultural assessment tools, such as the RUDAS, may help build competence and confidence when assessing and caring for people with different cultural and linguistic backgrounds. Additionally, professionals within dementia teams and nurses in home-based services may collaborate with GPs to closely monitor a patient and observe the ‘complexity’ of symptoms and behaviours over time – within the context of that person’s observed mental capacity – and maintaining a proactive dialogue with the person and/or the family. Further, as in all person-centred care, health professionals should be wary of stereotyping and generalizing groups through assumptions about needs based on ethnicity, such as mistakenly assuming some minority ethnic groups have extended families looking after them. Additionally, immigrants in Norway is a very heterogenic group and the needs of older European labor immigrants is not necessarily the same as those of labor immigrants or refugees from other continents. Finally, outreach activities aimed at immigrant organizations and more proactive health professionals may generate further awareness and acknowledgement of dementia as an illness rather than as a normal part of ageing or a condition associated with stigmatized explanatory models. Such an approach may engender more openness and motivate individuals as well as families to seek help when needed.

## Additional files

Additional file 1: Interview Guide Health Personnel (this guide exists in several versions, as the guide were partly adjusted to the ongoing data collection as well as the type of work place/health personnel being interviewed). (DOCX 17 kb)

Additional file 2: Focus Group Guide Health Personnel (this guide exists in several versions, as the guide were partly adjusted to the ongoing data collection as well as the type of work place/health personnel participating in the Focus Groups). (DOCX 16 kb)

## Abbreviations

AD: Alzheimer’s disease
FGD: Focus Group Discussion
GP: General Practitioners
IDI: In-Depth Interview
MMSE: Mini Mental State Examination
REC: Regional Ethical Committee
RUDAS: The Rowland Universal Dementia Assessment Scale
