# Supplementary material for: Dementia and immigrant groups: a qualitative study of challenges related to identifying, assessing, and diagnosing dementia
Source: BMC Health Serv Res. 2018 Nov 29;18:910. doi: 10.1186/s12913-018-3720-7 (PMC6267848; doi:10.1186/s12913-018-3720-7)
Supplement: Supplementary file 1 — Interview Guide Health Personnel (this guide exists in several versions, as the guide were partly adjusted to the ongoing data collection as well as the type of work place/health personnel being interviewed). (DOCX 17 kb) [file 12913_2018_3720_MOESM1_ESM.docx]

# Dementia and older immigrants – in depth interviews

INTRODUCTION

- Project's background and goals
- Information and confidentiality
- Audio Recording
- Consent Statement
- Timeframe

EXPERIENCES

- Can you say something about your experiences with older patients with immigrant background?
  - Communication / understanding
  - Relatives / family relationship
- Can you say something about your experiences with older immigrant patients with cognitive impairment (not yet diagnosed)?
  - Special needs / procedures / measures?
  - Language/communication/interpreter, involvement of relatives?
  - Patients and relatives' knowledge of and view of dementia?
  - Cultural-specific understandings/interpretations?
  - Use of assessment tools. Knowledge of RUDAS?
  - Referral to the specialist health service?
    - Where?
    - When/Why?
    - Collaboration?
  - Referral to other services?
    - Where?
    - When/Why?
    - Collaboration?
- Can you say something about your experiences with older immigrant patients after getting a dementia diagnosis?
  - Patients and relatives' knowledge of and view of dementia?
  - Patients and relatives’ need for information?
  - The role and commitment of family / relatives?
  - Collaboration with other actors / services?

ASSESSMENT

- How are assessment of dementia in immigrant patients (multilingual with other cultural backgrounds) normally carried out? (approach, assessment tools)
  - What are included in an assessment done by the GP?
  - What are included in the assessment done by specialists?
  - What are included in the assessment done by the dementia coordinator?
  - Which tests and assessment tools do you think should be used?
  - Who are normally referred/who do you think should be referred to specialists?

FOLLOW-UP AND COOPERATION

- How do you think this patient group should be followed up?
  - Which other services / actors are important to involve?
  - How should the cooperation and coordination between primary and secondary services be?
  - How to cooperate with relatives?
  - How to facilitate good communication / information?

KNOWLEDGE NEEDS

- What type of knowledge (general practitioner/specialist/health personnel) do you need to give good treatment and care to this group?
  - What do you need to know more about?
  - Where and in what form of should knowledge be available?

SUMMARY

- Other issues / topics that you want to say something about?
