# Supplementary material for: Dementia and immigrant groups: a qualitative study of challenges related to identifying, assessing, and diagnosing dementia
Source: BMC Health Serv Res. 2018 Nov 29;18:910. doi: 10.1186/s12913-018-3720-7 (PMC6267848; doi:10.1186/s12913-018-3720-7)
Supplement: Supplementary file 2 — Focus Group Guide Health Personnel (this guide exists in several versions, as the guide were partly adjusted to the ongoing data collection as well as the type of work place/health personnel participating in the Focus Groups). (DOCX 16 kb) [file 12913_2018_3720_MOESM2_ESM.docx]

# Dementia and Older Immigrants – Focus group guide

EXPERIENCES

- Can you start by discussing your experiences with older patients with immigrant background?
  - Communication / understanding
  - Relatives / family relationship
- Can you say something/discuss your experiences with older immigrant patients with cognitive impairment (not yet diagnosed)?
  - Special needs / procedures / measures?
  - Language/communication/interpreter, involvement of relatives?
  - Patients and relatives' knowledge of and view of (early) symptoms?
  - Cultural-specific understandings/interpretations?
  - Referral to the specialist health service?
  - Referral to other services?
- Can you say something about your experiences with older immigrant patients who get a dementia diagnosis?
  - Special needs for information about dementia in immigrant groups?
  - Patients and relatives' knowledge of and view of dementia?
  - Patients and relatives’ need for information?
  - The role and commitment of family / relatives?
  - Collaboration with other actors / services?

FOLLOW-UP AND COOPERATION

- How do you think this patient group needs to be followed up?
  - Other services / actors important to involve?
  - Cooperation / coordination between primary and secondary services?
  - Cooperation with relatives?
  - Facilitation of good communication / information?

KNOWLEDGE NEEDS

- What knowledge do you (in your different positions) need to give good treatment and care to this group?
  - What do you need to know more about?
  - Where and in what form should knowledge be available?

SUMMARY

- Other issues / topics that you want to say something about?
